# Artificial Intelligence Network Embedding, Entrepreneurial Intention, and Behavior Analysis for College Students’ Rural Tourism Entrepreneurship

**DOI:** 10.3389/fpsyg.2022.843679

**Published:** 2022-05-27

**Authors:** Zhonghui Kang

**Affiliations:** School of Culture and Communication, Guilin Tourism University, Guilin, China

**Keywords:** entrepreneurial intention, artificial intelligence network, entrepreneurial willingness, rural tourism, college students’ entrepreneurial mental resilience

## Abstract

To promote the development of the rural economy and improve entrepreneurship education in colleges and universities, college students’ willingness and behavior toward rural tourism entrepreneurship were investigated in this study. First of all, based on the previous research results, the influencing factor model was determined for college students’ entrepreneurial intention. Second, a questionnaire survey was made to collect data from a university in Xi’an City. Finally, the artificial neural network (ANN), improved by a genetic algorithm (GA) based on an artificial intelligence network, was used to study the relationship between college students’ entrepreneurial intention and behavior, and the simulation was carried out on MATLAB2013b software. The results show that the average evaluation accuracy is 81.13% for 60 groups of data using the unmodified back propagation neural network (BPNN) algorithm, while the average evaluation accuracy is 92.17% for the BPNN algorithm improved and optimized by GA, with an ascent of 11.04%. Therefore, the BPNN algorithm improved and optimized by GA is better than the unmodified BPNN algorithm; It is also feasible and effective in the analysis of influencing factors of college students’ entrepreneurial intention and behavior. The research provides a basis for colleges and universities to carry out entrepreneurship education on a large scale and to cultivate their innovative talents.

## Introduction

In 2017, the word “rural” became popular rapidly and began to attract public attention. In recent years, the phenomenon of rural economic recession has become more obvious in China. Revitalizing rural economy and culture has become the focus of all sectors of society. These problems are highlighted in the urgent need for various policy support, efforts, and cooperation. Every effort should be made to promote the development of a socialist harmonious society and take rural tourism as the main field and main way to realize rural development (Chen, 2019).

At present, the number of college graduates is surging, resulting in great employment pressure. For this reason, colleges and the government encourage college students to start businesses, which can not only alleviate the employment pressure but also promote the development of the rural economy. College students’ entrepreneurship requires strong support from campuses and the government, as well as relevant entrepreneurial education. Therefore, there are many studies on college students’ entrepreneurial willingness and behavior. Wu et al. (2019) and Wu and Song (2019) discussed the use and satisfaction of social media in entrepreneurship courses from the perspective of learners, while the respondents were not college students, but more entrepreneurs. It is very important to explore and find the driving factors behind entrepreneurial intention for entrepreneurial education and entrepreneurial practice. Wu et al. (2019) took the MBA students of Tianjin University as a sample and analyzed the relationship between the dark Trinity, entrepreneurial self-efficacy, and entrepreneurial intention. Suratno et al. (2021) explored other predictors of students’ entrepreneurial intention, such as family economic education, peer groups, and economic literacy. The results show that family economic education and peer groups are positively correlated with students’ economic literacy and entrepreneurial intention. Based on social network and organizational learning theory, Wu et al. (2020) utilized the intermediary effect of entrepreneurial learning and analyzed the role of entrepreneurial learning (exploratory and developmental) in the relationship between internal and external networks and enterprise growth performance in a dynamic environment. Nguyen and Duong (2021) described a data set to explore the impact of perceived education support on entrepreneurial self-efficacy, entrepreneurial attitude, subjective norms, perceived behavior control, and entrepreneurial intention. A lot of literature are released focusing on the influencing factors of entrepreneurship education and entrepreneurial intention (Douglas et al., 2021) because entrepreneurial intention plays a vital role in whether college students choose entrepreneurship. It is very important to find out the influencing factors that play a key role in entrepreneurial intention (Liao, 2020).

Few scholars use artificial neural network (ANN) technology, especially the ANN technology optimized by genetic algorithm (GA), to analyze and study the entrepreneurial intention and behavior of college students. What is the current status of college students’ willingness to return to their hometown for business startups? Which college students are more willing to return to their hometown for business startups? What are the influencing factors of college students’ willingness to return to their hometown for business startups, and what are their mechanisms? In response to these questions, a questionnaire was designed. A questionnaire was developed, which sampled from a university in Xi’an, collected data, processed the data, and studied the relationship between College Students’ entrepreneurial intention and entrepreneurial behavior with the artificial neural network improved by GA. This conclusion can provide theoretical support for China’s colleges and universities to widely carry out entrepreneurship education and improve the level of entrepreneurship education. The innovation is to use the ANN technology optimized by GA to analyze and study the entrepreneurial intention and behavior of college students.

## Materials and Methods

### Factors and Models of College Students’ Entrepreneurial Intention

Generally, when studying the entrepreneurial intention and behavior of college students, a model will be established in advance, and the research will be carried out based on the established model, which can make the research ideas clear. In recent years, researchers have used three models for the factors affecting entrepreneurial intention (Iwu et al., 2021; Karimi and Saghaleini, 2021) theoretical model of planned behavior, entrepreneurial event model, and two-factor model.

#### Theoretical Model of Planned Behavior

**FIGURE 1:**
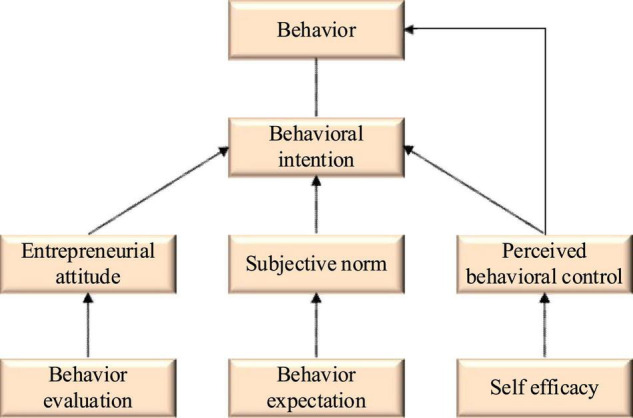
Theoretical model of planned behavior.

In social psychology, the planned behavior theory is considered to be the most influential Attitude-Relationship theory, so it is very appropriate to be used in the study of entrepreneurial intention (Luo et al., 2018). The theoretical model of planned behavior includes three elements: attitude, subjective norms, and perceived behavior control. Attitude refers to the entrepreneurs’ evaluation of this entrepreneurial behavior in entrepreneurship, which can be either positive or negative (Mensah et al., 2021). Chen (2019) and Nithya and Lakshmi (2019) believed that subjective norms are the expectations of entrepreneurs who are influenced by the views of their parents, friends, and relatives. As for perceived behavior, control is a kind of control ability that a person shows from perception to the implementation of a certain behavior, which is dominated by behavior intention, and attitude. Subjective norms and perceived behavior control will have a certain impact on behavior intention. Figure 1 displays the structure of the theoretical model of planned behavior.

#### Entrepreneurial Event Model

**FIGURE 2:**
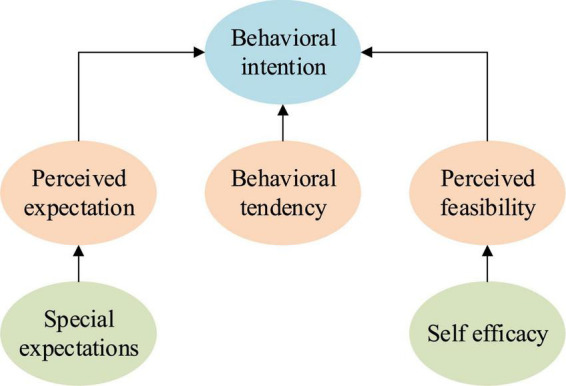
Entrepreneurial event model.

The entrepreneurial event model mainly consists of behavior tendency, perceived expectation, and perceived feasibility. Among these three aspects, it is the highest of the correlation between behavior tendency and willingness. The essence of the behavioral tendency is the probability that such behavior may occur, while the perceived expectation is the expectation brought by individual perceived behavior. As for perceived feasibility, it is based on the evaluation of self-efficacy, and then makes a reasonable and feasible prediction of the feasibility of behavior (Neto et al., 2020). Figure 2 illustrates the structure of the entrepreneurial event model.

By comparing the theoretical model of planned behavior with the entrepreneurial event model, results show that the two models are similar in many places. For example, the variable of perceived expectation in the entrepreneurial event model can explain the variable of entrepreneurial attitude in the theoretical model of planned behavior; moreover, some variables can be reflected by self-efficacy in both models.

#### Two-Factor Model

**FIGURE 3:**
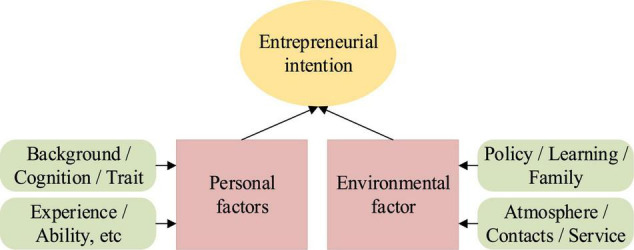
Two-factor model.

The two-factor model includes two categories: personal factors and environmental factors. Personal factors consist of internal factors, such as personal background, experience, cognition, and ability, while environmental factors refer to some external factors, such as policy support, interpersonal relationship, school education, and family and social support (Nguyen and Duong, 2021). Figure 3 presents the features of the two-factor model.

**FIGURE 4:**
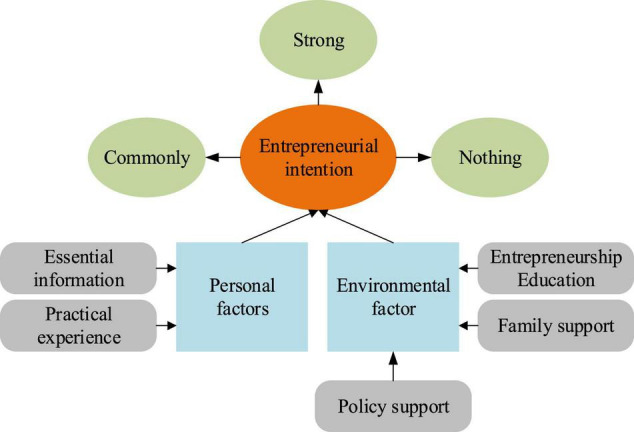
Influencing factor model of college students’ entrepreneurial intention.

The research was carried out on college students. Figure 4 indicates the influencing factor model of college students’ entrepreneurial intention.

### Variables and Hypotheses of the Experiment

In this study, college students were selected as the survey object, and the variables were set according to the college students, mainly including basic information, practical experience, entrepreneurship policy, school entrepreneurship education, and family entrepreneurship support. The specific variables are as follows:

(1) Basic information: Usually, men have higher entrepreneurial intentions than women. Students with college or undergraduate education will have higher entrepreneurial intentions than students with a graduate degree or above. Moreover, the entrepreneurial intention reflected by different majors is also different. Generally, students of economics and management and finance may have higher entrepreneurial intentions, because the professional knowledge learned by these students will be helpful for entrepreneurship. If someone in their family has relevant entrepreneurial experience, their entrepreneurial willingness will be higher (Saptono et al., 2021).
(2) Practical experience: Generally speaking, students who have served as cadres in the school have the strong organizational ability, help in entrepreneurship, and have higher entrepreneurial willingness; students who have participated in some entrepreneurial activities organized by the school will have some understanding of entrepreneurship, so their entrepreneurial willingness will be higher.
(3) Entrepreneurship policy: If some students have carefully studied the entrepreneurship policy or have a better understanding of the policy, their willingness will be higher than those who do not understand the entrepreneurship policy.
(4) School entrepreneurship education: Commonly, students will be exposed to school entrepreneurship education in universities, but not all students can absorb entrepreneurship education. Some students may give a high evaluation to school entrepreneurship education, which shows that school entrepreneurship education has achieved good results; these students will have relatively high entrepreneurial willingness (Vodă and Florea, 2019). Wu and Wu (2017) and Wu and Song (2019) made a detailed analysis of entrepreneurship education, focusing on the impact relationship between entrepreneurship education and entrepreneurial intention.
(5) Family entrepreneurship support: Normally, entrepreneurship is inseparable from family support. If some students have relevant contacts at home or material and spiritual help, it will be easier for them to start a business, so they will have a higher willingness to start a business.

### Artificial Intelligence Network

#### Back Propagation Neural Network

**FIGURE 5:**
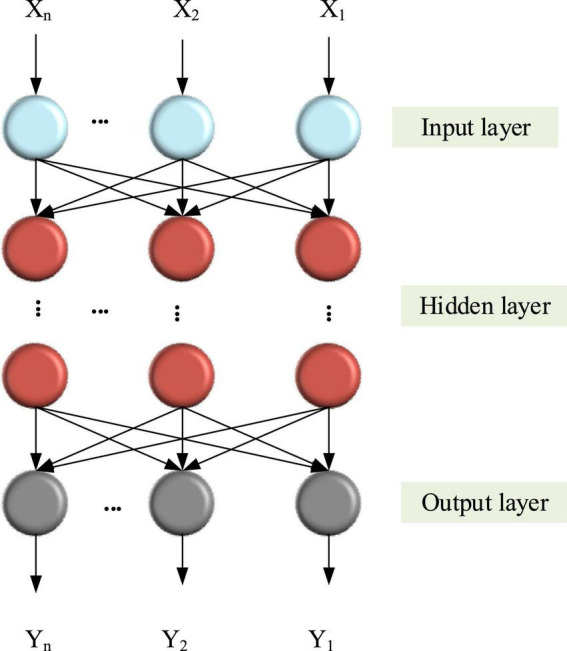
Structure of BPNN.

Back propagation neural network adds a backward propagation algorithm to the structure of feed-forward network. It not only has input and output nodes but also has one or more hidden layer nodes. It is a one-way propagation multilayer forward network (Wu and Wu, 2017). The basic idea of this method is to minimize the mean square error between the actual output and the actual output by using the gradient search technology, and then use the gradient search technology to perform gradient search on the output node (Zhang et al., 2020, 2021). Figure 5 indicates the structure of BPNN.

The input mode vectors were set as:


(1) $$X^{\text{k}}={\left(x_{1}^{k},x_{2}^{k},\cdots ,x_{n}^{k}\right)}^{T}\left(k=1,2,\cdots ,m\right)$$

where n represents the number of units of the input layer; m represents the number of learning mode pairs.

Equation 2 denotes the calculation of the desired output vector of the corresponding input mode.


(2) $$Y^{\text{k}}={\left(y_{1}^{k},y_{2}^{k},\cdots ,y_{q}^{k}\right)}^{T}$$

where q represents the number of output layer units.

Equation 3 shows the calculation of the net input vector corresponding to the middle hidden layer.


(3) $$S^{\text{k}}={\left(s_{1}^{k},s_{2}^{k},\cdots ,s_{p}^{k}\right)}^{T}$$

The output vector can be expressed as:


(4) $$B^{\text{k}}={\left(b_{1}^{k},b_{2}^{k},\cdots ,b_{p}^{k}\right)}^{T}$$

where p represents the number of hidden layer units.

The net input vector of the output layer can be expressed as:


(5) $$L^{\text{k}}={\left(l_{1}^{k},l_{2}^{k},\cdots ,l_{q}^{k}\right)}^{T}$$

Then, there is the actual output vector.


(6) $$C^{\text{k}}={\left(c_{1}^{k},c_{2}^{k},\cdots ,c_{q}^{k}\right)}^{T}$$

The neuron thresholds of the hidden layer and the output layer are supposed as α and β, whose expressions are as follows:


(7) $$\alpha =\left\{\alpha_{0},\cdots ,\alpha_{S-1}\right\}$$

(8) $$\beta =\left\{\beta_{0},\cdots ,\beta_{S-1}\right\}$$

**FIGURE 6:**
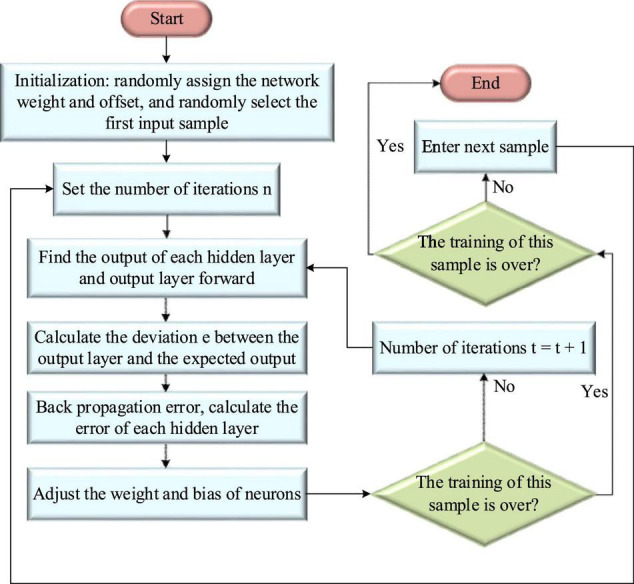
BPNN learning algorithm flow.

Figure 6 shows the specific algorithm flow of BPNN.

In the practical application of BPNN, there are many pros and cons, as shown in Tables 1, 2.

**TABLE 1:** Specific assumptions.

Grade	Specific assumptions
H1	Students majoring in finance and economics have higher entrepreneurial willingness.
H2	Boys are more willing to start a business than girls (Wu et al., 2019).
H3	Urban residence has higher entrepreneurial desire than rural residence.
H4	College students with bachelor degree are more willing to start a business.
H5	Students whose families have entrepreneurs have higher entrepreneurial intention (Wu et al., 2020).
H6	Students who have served as class cadres have higher entrepreneurial willingness.
H7	College students who have participated in school entrepreneurship activities have stronger entrepreneurial willingness.
H8	College students who know about entrepreneurship policies have higher entrepreneurial willingness.
H9	College students with good implementation of entrepreneurship education have stronger entrepreneurial willingness.
H10	College students with good family entrepreneurship support have stronger entrepreneurial willingness.

**TABLE 2:** Pros and cons of BPNN.

Pros	Cons
Non-linear processing can be carried out.	It has uncertainty of initial weight and threshold.
It has good fault tolerance.	The network is easy to fall into local minimum.
It has good function approximation and pattern classification.	The selection of learning rate is lack of effective methods.
It is easy to implement.	There is no effective method to determine the number of hidden layer neurons.

#### Genetic Algorithm

**FIGURE 7:**
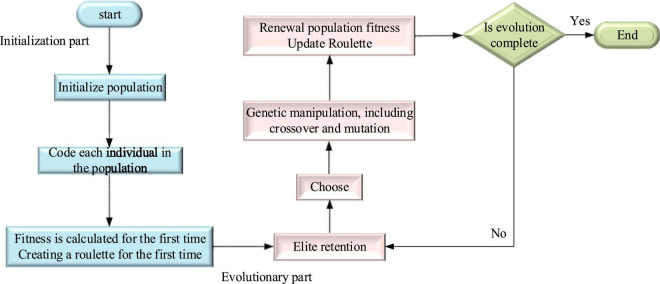
Flow of GA.

Genetic algorithm (GA) is also called an evolutionary algorithm. Its main advantage is that it does not have restrictions on function continuity and derivation, and can directly operate on structural objects. Therefore, GA is different from other algorithms for finding optimal solutions (Zhu et al., 2021). Another advantage is that GA adopts a probabilistic optimization method, which can automatically obtain rules that do not need to be determined and can adaptively adjust the search direction (Wang et al., 2021). Figure 7 displays the flow of GA.

**FIGURE 8:**
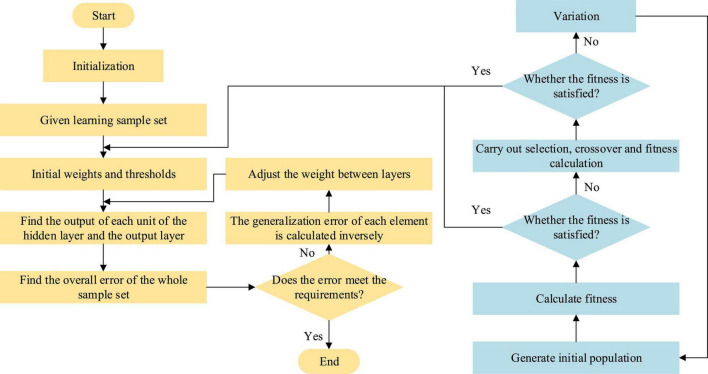
Flow of BPNN optimized by GA.

Genetic algorithm has been widely used in many scientific fields because it has strong robustness to problems. It does not depend on the specific field of the problem, gradient information, or other auxiliary knowledge, but provides a general framework for solving complex system problems (Zhu and Shang, 2021). Because of these advantages, GA can be used to effectively solve the shortcomings of BPNN. Therefore, combined with the advantages of GA, the BPNN sees its optimization and improvements, and the study is augmented by the algorithm in the analysis of the relationship between college students’ entrepreneurial intention and behavior. Figure 8 signifies the process of optimizing BPNN by GA. In this process, the most critical step is to take the global optimal solution satisfying the optimization objective obtained by GA as the initial weight and threshold of the neural network, which not only reduces the complexity of the training process but also optimizes BPNN in time.

(1) Calculation of the fitness. The search goal of GA is to obtain the network weight and threshold that minimizes the sum of squares of network errors in all evolutionary generations and set the fitness function as the reciprocal of each learning error. Equation 9 denotes the learning error and Eq. 10 indicates the fitness function.


(9) $$E=\frac{\sum_{k=1}^{p}\sum_{j=1}^{l}\left(y_{j}^{k}-o_{j}^{k}\right)}{2}$$

(10) $$fitness=\frac{1}{E}$$

where E represents the learning error, *p* refers to the number of training samples, *l* stands for the number of output nodes, and $y_{j}^{k}-o_{j}^{k}$ means the error of the k_*th*_ sample relative to the j_*th*_ output node.

#### Parameter Determination of Back Propagation Neural Network

Too many or too few neurons may degrade the performance of the algorithm. At present, there are several methods to determine the number of neurons:


(11) $$\sum_{j=0}^{n}c_{n_{j}}^{j}>m,j\in \left[0,n\right]$$

In Eq. 11, *m* represents the number of samples, *n*_*j*_ means the number of neurons in the hidden layer, and *n* refers to the number of input units.


(12) $$n_{1}=\sqrt{n+m}+a,a\in \left[1,10\right]$$

In Eq. 12, *n*_1_ denotes the number of neurons in the hidden layer, *n* stands for the number of neurons in the input layer, *m* equals to the number of neurons, and *a* is a constant in the range of 1 to 10.


(13) $$N_{1}=log2^{n}$$

In Eq. 13, *N*_1_ represents the number of neurons in the hidden layer and *n* refers to the number of neurons in the input layer.

The learning speed determines the convergence speed of BPNN, and they are in direct proportion. The learning rate value is selected as 0.01 according to the empirical value, which involves the output of expected and actual values, as shown below.


(14) $$H_{i}=f\sum_{i=1}^{n}\omega_{ij}x_{i}-\alpha_{j}),j=1,2,\cdots ,l$$

In Eq. 14, *H*_*i*_ refers to the desired output value, ω_*ij*_ stands for the connection weight between the input layer and the hidden layer, and *f* represents the hidden layer function. Parameter *l* denotes the number of neurons in the hidden layer. And α means the threshold.


(15) $$O_{k}=\sum_{i=1}^{i}H_{j}\omega_{jk}-b_{k}),k=1,2,\cdots ,m$$

In Eq. 15, *O*_*k*_ represents the actual output value, *ω_*jk*_* refers to the connection weight between the hidden layer and the output layer, and *b*_*k*_ stands for the threshold.

It is necessary to standardize the data (Song et al., 2018) to make it comply with the data standard processed by BPNN. In the experiment, the maximum value method and minimum value method are used to standardize the data. The calculation method is as follows.


(16) $$x=\frac{x_{ij}-\min x_{ij}}{\max x_{ij}-\min x_{ij}},i=1,2,\cdots ,n,j=1,2,\cdots ,m$$

where *x* represents the normalized data value, *x*_*ij*_ denotes the unprocessed data value, *min*⁡*x*_*ij*_ refers to the minimum data value, and *max*⁡*x*_*ij*_ stands for the maximum data value.

### Weight Calculation of Various Indicators

To obtain the relationship between neurons, the BPNN optimized by GA is used for learning. Then, the basic information, time experience, policy support, entrepreneurship education, and weights of family support are measured. The specific steps are as follows:

The first step is to calculate the correlation significance coefficient of each index *r*_*ij*_, the calculation is as follows:


(17) $$r_{ij}=\sum_{k=1}^{p}w_{ki}\left(1-e^{-x}\right)/\left(1+e^{-x}\right)$$

(18) $$x=w_{jk}$$

where, *j* refers to the output layer unit and *j* = 1,2,…, n; *k* means the hidden layer unit and *k* = 1,2,…, p; *w*_*jk*_ denotes the weight between *j* and *k*; *w*_*ki*_ stands for the weight between *i* and *k*.

The second step is to calculate the relevant index of each index *R*_*ij*_, the calculation is as follows:


(19) $$R_{ij}=\left|\left(1-e^{-y}\right)/\left(1+e^{-y}\right)\right|$$

(20) $$y=r_{ij}$$

The third step is to calculate the weight of each index and its accounting model *S*_*ij*_ is:


(21) $$S_{ij}=\frac{R_{ij}}{\sum_{i=1}^{m}R_{ij}}$$

where *R*_*ij*_ stands for the correlation index, *i* denotes the output unit, and i = 1,2,…, m; the weight of each index can be solved through the above three steps.

### Reliability and Validity Analysis of the Scale

Generally, reliability and validity analysis should be carried out before processing the collected data. Reliability analysis refers to testing the authenticity of data through Cronbach’s alpha coefficient. The closer the coefficient value is to 1, the higher the credibility of the scale, which means the better the reliability. Generally, it is considered acceptable if the coefficient value is 0.7. The validity is usually tested by Kaiser-Meyer-Elkin (KMO) and Bartlett sphere. The judgment criteria are as follows: (1) those whose KMO ≥ 0.8 are most suitable for factor analysis; those whose 0.7 ≤ KMO < 0.8 can also be applied to factor analysis; those whose 0.6 ≤ KMO < 0.7 are not suitable for factor analysis and not recommended; and those whose KMO < 0.6 are not suitable for factor analysis; (2) whether it is significant of the probability of Bartlett’ spherical test result; (3) the cumulative interpretation variance was greater than 60%; and (4) the factor load is greater than 0.5 and the cross factor load is less than 0.4.

## Results and Discussion

### Descriptive Statistics of Samples

The research group comprises the students of the Xi’an University of Technology. A total of 500 questionnaires are distributed, 490 are recovered, some incomplete questionnaires are deleted, and the remaining 470 questionnaires are valid with an effective recovery rate of 94%. Table 3 illustrates the specific statistical data of college students.

**TABLE 3:** Sample statistics.

Variables	Specific situation	Proportion
Major	Finance and management	31.5%
	Other	68.5%
Gender	Male	47.4%
	Female	52.6%
Household register	Countryside	41.7%
	City	58.3%
Education	Technical undergraduate	86.9%
	Bachelor degree or above	13.1%
Class cadres	Yes	65.2%
	No	34.8%
Family entrepreneurship	Yes	45.6%
	No	54.4%
Participation in entrepreneurial activities	Yes	22.1%
	No	77.9%

Table 3 signifies that, in terms of gender, girls account for 52.6% of the total sample, while boys account for only 47.4%; in terms of academic qualifications, the proportion of college students is still relatively large, which is 86.9%; the difference in the family entrepreneurship is relatively balanced; students who have served as class cadres account for 65.2%, whose portion is relatively large. Among the place of domicile, urban college students are more willing to return to their hometown to start a business than rural college students, with a difference of 16.6%. In short, the willingness of college students to start a rural tourism business has a large individual characteristic difference. Students majoring in finance and economics and management are more willing to start a business (*p* < 0.01). Boys are more willing to start a business than girls (*p* < 0.01). The place of urban domicile is more willing to start a business than the place of rural domicile (*p* < 0.01). College students with a degree are more willing to start a business (*p* < 0.01). Students whose family has someone to start a business are more willing to start a business (*p* < 0.01). College students who have participated in school entrepreneurship activities have a stronger entrepreneurial willingness (*p* < 0.01). College students who understand entrepreneurial policies have a higher entrepreneurial willingness (*p* < 0.01). College students who have implemented entrepreneurship education in schools have a stronger entrepreneurial willingness (*p* < 0.01). Students with good family entrepreneurship support have a stronger entrepreneurial intention (*p* < 0.01). Therefore, the above hypothesis is verified.

### Reliability and Validity Analysis of Samples

SPSS19.0 software is used to test the reliability of the data of each scale. The Bartlett sphericity test, KMO inspection, and test values of the Alpha coefficient are shown in Table 4.

**TABLE 4:** Reliability and validity data.

KMO inspection value	0.854	
Bartlett spherical test value	Approx. Chi-Square	5899.842
	Df	260
	Sig.	0.000
Alpha coefficient	0.882	

The KMO value and Alpha coefficient of eight aspects set in this questionnaire are 0.854 and 0.882, respectively, close to 0.9, which is very suitable for factor analysis, indicating that this questionnaire has high reliability and validity. Through Bartlett spherical test, the statistical significance *P*-value is 0.000, less than 0.001, so these eight aspects are suitable for factor analysis.

### Evaluation and Statistics of Entrepreneurial Environment

**FIGURE 9:**
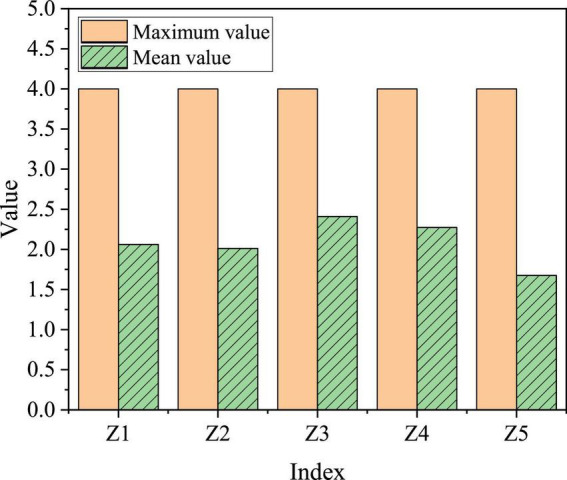
Evaluation and statistics of college students on school entrepreneurship policy.

The evaluation of the entrepreneurial environment is mainly carried out through students’ scoring. The form of the scoring is a five-point system. From 1 to 5, there are five grades: very poor, general, medium, good, and very good. The entrepreneurship policies of government are divided into government approval procedures, financing channels, tax incentives, capital subsidies, and project guidance, which are expressed in Z1 to Z5, respectively. Figure 9 signifies the specific evaluation results.

The maximum value of entrepreneurship policy is 4, the lowest average value is Z5 (government project guidance), which is only 1.6752, and the highest average is Z3 (tax preference), which is 2.4101. Generally speaking, the average values of these five variables are about 2, which is between “moderate” and “medium.”

**FIGURE 10:**
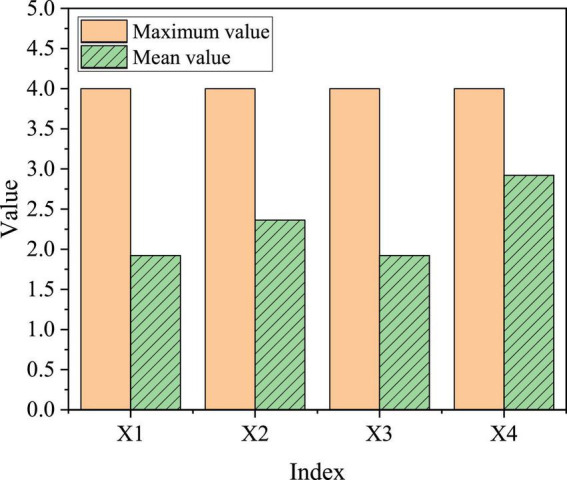
Evaluation and statistics of college students’ entrepreneurial environment.

The school’s entrepreneurship environments were divided into four parts: entrepreneurship courses in colleges, lectures of successful entrepreneurs, entrepreneurship training, and entrepreneurship competition, which are expressed in X1 to X4, respectively. Figure 10 presents the specific evaluation results.

The maximum score for the schools’ entrepreneurial environment is 4, the highest average is X4 (the school holds an entrepreneurial competition), which is 2.9216, while the average values of X1 and X2 are the lowest, both of which are 1.9216. On the whole, the average value is between 1.9 and 2.9, which is about the medium. The clearer the conditions of the entrepreneurship policy, the more attractive it will be for college students to return to their hometowns to start a business. Therefore, the entrepreneurial environment has a significant impact on the willingness of college students to return to their hometowns to start a business.

**FIGURE 11:**
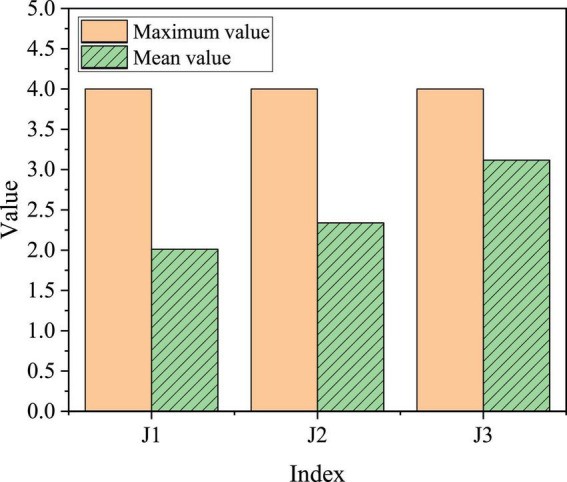
Evaluation and statistics of college students for entrepreneurship support from family.

Family entrepreneurship support is divided into three parts: family networking help, financial support, and spiritual support, which are expressed in J1 to J3, respectively. Figure 11 demonstrates the specific evaluation results.

J3 (family spirit support) has the highest mean value in family entrepreneurship support, with a value of 3.12, and J1 (family network help) has the lowest mean value, with a value of 2.01. Generally speaking, the mean value is between 2 and 3.1, that is, between medium and good. Family characteristics have an impact on college students’ willingness to return to their hometowns to start a business. Parents’ entrepreneurial behavior will affect their own children’s entrepreneurial willingness and behavior. College students whose parents have started a business or own their own company may take fewer detours when returning to their hometowns to start a business because they have successful entrepreneurial models or useful entrepreneurial experiences around them. Their willingness will naturally be stronger.

### Statistical Analysis of College Students’ Entrepreneurial Willingness

**FIGURE 12:**
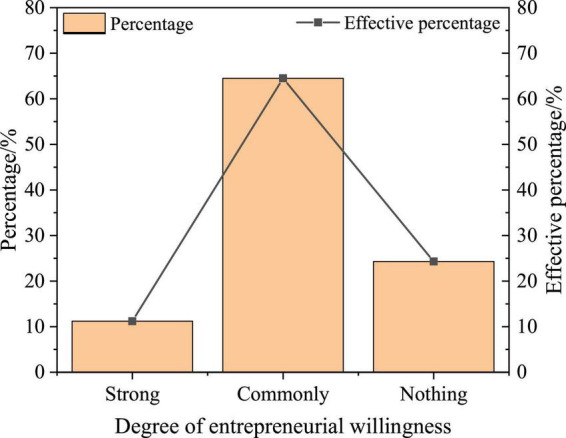
Statistics of the entrepreneurial willingness of college students.

**FIGURE 13:**
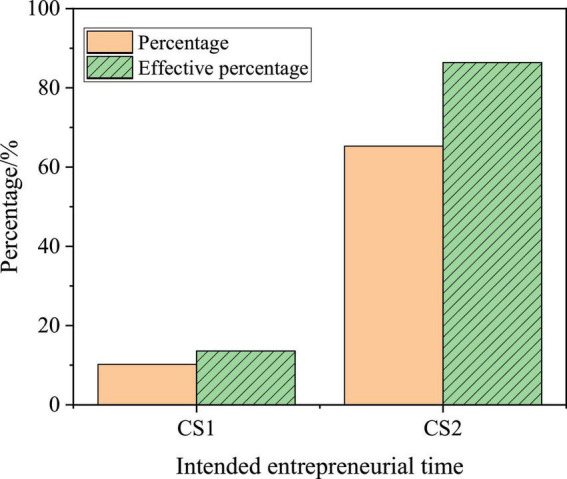
Statistics of the time when college students intend to start a business.

**FIGURE 14:**
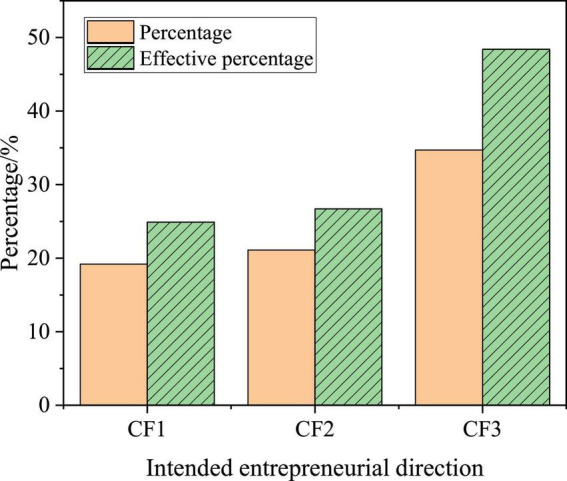
Statistics of college students’ entrepreneurial direction.

The willingness to start a business can be analyzed from three aspects: the degree of willingness, the time of willingness to start a business, and the direction of willingness to start a business, as shown in Figures 12–14.

According to Figure 12, 11.2% of college students have strong entrepreneurial willingness, 64.5% have common entrepreneurial willingness, and 24.3% have no entrepreneurial willingness, which shows that college students’ entrepreneurial willingness is relatively high, but most of them are still at the common level.

The division is made on the time when college students intend to start a business as: CS1, which means to start a business without working after graduation; and CS2, which means to start a business after graduation. Figure 13 indicates the specific statistical results.

Most college students choose to work before starting a business, accounting for 86.4%; while only 13.6% of college students choose to start a business directly after graduation. The reason for this is that college students do not have the support of resources and funds and lack experience, so most students do not intend to start a business directly after graduation.

In terms of entrepreneurial direction, some students choose the industries related to their major, while other students choose those industries unrelated to their major, and a few students are still uncertain about their entrepreneurial direction. They think both the related industries and the unrelated ones are fine. The choices of the college students can be represented by CF1, CF2, and CF3, respectively, whose specific statistical results are shown in Figure 14.

According to Figure 14, 48.4% of college students are not sure about their entrepreneurial direction, which is determined according to the specific situation after their graduation, while 21.1% of college students intend to start a business related to their majors in the school, which shows that the professional course knowledge learned in the school will still have some impact on the entrepreneurial direction, but the impact is not great.

### Error Rate and Performance of Training for Improved Back Propagation Neural Network

**FIGURE 15:**
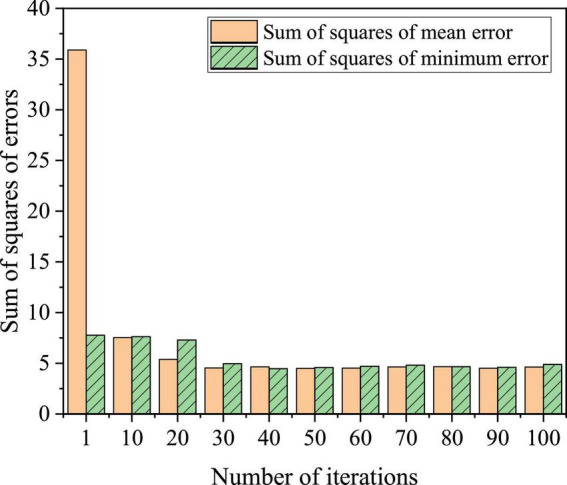
Sums of squares of errors.

**FIGURE 16:**
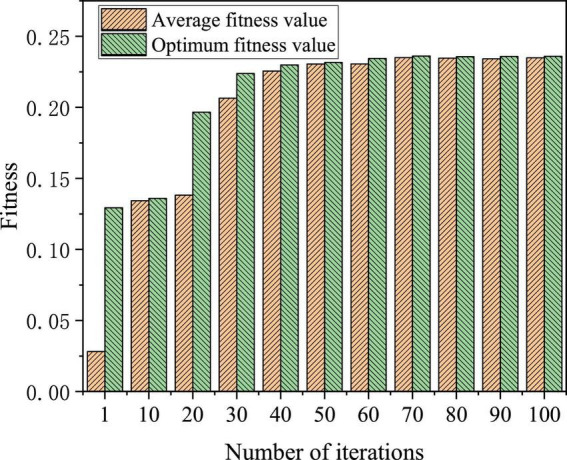
GA-BPNN fitness results.

The first 410 samples collected were used as training samples and the last 60 samples were used as test samples. The simulation experiments were carried out on MATLAB2013b. The training error performance comparison of the BPNN improved by GA is shown in Figure 15, and the fitness comparison is shown in Figure 16.

The convergence speed of the square sum of BPNN error improved and optimized by GA is very fast before the 5th generation of the algorithm, the convergence speed is relatively stable between the 5th generation and 20th generation, and the convergence speed is relatively slow between the 20th generation and 30th generation. After the 35th iteration, the sums of squares of errors reach a stable state in BPNN improved and optimized by GA, which shows that the BPNN model improved and optimized by GA can quickly realize global optimization.

The convergence speed of the BPNN fitness function improved and optimized by GA is relatively fast before the 10th generation, reaching a relatively gentle state between the 10th generation and 20th generation, and reaching a stable state after 40 iterations. The adaptability is relatively high for the BPNN model improved and optimized by GA. In a word, the training and prediction performance of BPNN improved and optimized by GA is determined by its internal mechanism. Evaluating the prediction accuracy and adaptability, the training is effective and robust of the BPNN model improved and optimized by GA.

### Performance Comparison Between Back Propagation Neural Network and Improved Back Propagation Neural Network

**FIGURE 17:**
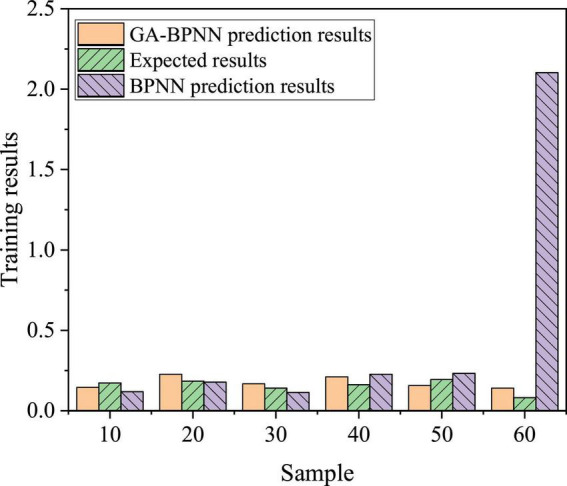
Prediction results.

The performance of the model was verified by BPNN, and the samples were simulated and compared. Figure 17 denotes the comparison of the prediction results.

The prediction results deviate greatly among individual samples of the BPNN algorithm, but the prediction results are relatively stable of the BPNN algorithm improved and optimized by GA. Compared with the unmodified BPNN, the BPNN algorithm improved and optimized by GA has a better performance effect.

The performance comparison between BPNN and BPNN improved and optimized by GA shows that the average evaluation accuracy is 81.13% of 60 groups of data of BPNN improved and optimized by GA, while the average evaluation accuracy is 92.17% of BPNN improved and optimized by GA, with an increase of 11.04%. BPNN improved and optimized by GA is better than BPNN without improvement. Besides, it shows that the BPNN algorithm improved and optimized by GA is feasible and effective in the analysis of influencing factors of college students’ entrepreneurial willingness and behavior.

## Conclusion

In recent years, China’s economy has developed rapidly, but the rural economy is still backward. To strike a balance between urban and rural areas, the government encourages college students to start businesses in their hometowns, to promote the rural economy and realize their values. In this study, a questionnaire was set up, and SPSS 19.0 software was used to test the reliability of the data of each scale, and sampling, data collection, and statistical analysis were carried out. The ANN improved by GA was used to study the relationship between college students’ entrepreneurial intention and behavior, and the simulation was carried out on MATLAB.

The results show that most college students will choose to work for a while after graduation, accounting for 86.4%, while only 13.6% of college students will choose to start a business directly after graduation. The simulation results show that the training and prediction performance of BPNN improved and optimized by GA is determined by its internal mechanism. Considering the prediction accuracy and adaptability, the training of the BPNN model improved and optimized by GA is effective and robust. The convergence speed of the BPNN fitness function improved and optimized by GA is relatively fast before the 10 iterations and it reaches a relatively gentle state between the 10 and 20 iterations. It can reach a stable state after 40 iterations. It can be seen that the adaptive ability of the BPNN model improved and optimized by GA is relatively high. Besides, it shows that the BPNN algorithm improved and optimized by GA is feasible and effective in the analysis of influencing factors of college students’ entrepreneurial intention and behavior. The research provides a basis for colleges to carry out entrepreneurship education on a large scale and cultivate innovative talents.

However, there are still some deficiencies. First, the number of samples collected is limited and only college students were sampled, so the survey is not comprehensive. In the later stage, more effort will be put into the study of college students who have graduated after 2 or 3 years. Second, the performance of the BPNN model improved and optimized by GA is better, and despite poor local search ability and slow search speed, GA should be optimized in future research.

## Data Availability Statement

The raw data supporting the conclusions of this article will be made available by the authors, without undue reservation.

## Ethics Statement

The studies involving human participants were reviewed and approved by the Guilin Tourism University Ethics Committee. The patients/participants provided their written informed consent to participate in this study. Written informed consent was obtained from the individual(s) for the publication of any potentially identifiable images or data included in this article.

## Author Contributions

The author confirms being the sole contributor of this work and has approved it for publication.

## Conflict of Interest

The author declares that the research was conducted in the absence of any commercial or financial relationships that could be construed as a potential conflict of interest.

## Publisher’s Note

All claims expressed in this article are solely those of the authors and do not necessarily represent those of their affiliated organizations, or those of the publisher, the editors and the reviewers. Any product that may be evaluated in this article, or claim that may be made by its manufacturer, is not guaranteed or endorsed by the publisher.
